# Quality Improvement Focused Morbidity and Mortality Rounds: An Integrative Review

**DOI:** 10.7759/cureus.12146

**Published:** 2020-12-18

**Authors:** Kayla P Churchill, Justin Murphy, Nick Smith

**Affiliations:** 1 Obstetrics and Gynecology, Faculty of Medicine, Eastern Health Memorial University, St. John's, CAN; 2 Orthopedic Surgery, Memorial University of Newfoundland, St. John's, CAN; 3 Orthopedic Surgery, Faculty of Medicine, Eastern Health Memorial University, St. John's, CAN

**Keywords:** healthcare quality, patient safety, medical education, morbidity and mortality

## Abstract

Morbidity and mortality conference (MMC) is a century-old tradition in medicine that was initially primarily focused on the review of surgical outcomes and errors. In recent years, the value of MMC in quality improvement (QI) and patient safety initiatives has been realized and incorporated into the MMCs of some disciplines and institutions. Despite this, there is a need for a standardized structure of MMC that emphasizes both QI and patient safety.

The purpose of this integrative review is to synthesize the literature on MMC structure that is reflective of QI and patient safety.

An integrative literature search was carried out using PubMed and MEDLINE. Abstracts were reviewed and non-relevant articles were excluded. Exclusion criteria were no mention of MMC, analysis of specific case, no focus on QI or patient safety, and non-English language.

A total of 21 articles were identified for review. Articles were reviewed in their entirety for content regarding structuring of the MMC to reflect and further develop QI and patient safety. The follwing three themes emerged that were consistently identified as being important for restructuring MMCs: (1) the importance of careful case selection, (2) the format of discussion during the conferences, and (3) the action plans reflecting QI initiatives derived from the conferences.

The review suggests that one standardized method of MMC implementation that encompasses the three pivotal themes should be developed. Further research needs to focus on instituting measures of effectiveness for the new MMC model.

## Introduction and background

Morbidity and mortality rounds or conferences originated in the early 1900s and were developed initially by the surgical disciplines to review surgical errors. Ernest Amory Codman, an American surgeon, laid the foundations of the morbidity and mortality conference (MMC). In addition to emphasizing the importance of review of outcomes in medicine for educational purposes, he championed the idea of an end-result card, which would outline the issues and errors identified in the case and would be subject to follow-up. He also suggested that a committee be employed to follow these results. However, this idea was met with resistance from the people of the medical community who were reluctant to involve people outside the community itself [1]. MMC eventually expanded further to encompass all of the disciplines within medicine [2].

It was not until many years later that the value of MMC with respect to quality improvement (QI) and patient safety was realized. In recent years, MMCs have been identified as a tool to promote and engage in QI and patient safety, in addition to its well-established educational piece [3]. Over the decades, the ability to identify patient safety issues and medical errors in the form of MMC and subsequent QI initiatives has been included in objectives for training by various governing bodies of medicine, including the Royal College of Physicians and Surgeons of Canada [4]. Despite this, MMCs differ in organization and execution between, and even within, disciplines, with most having no standard format [2]. In order to change the safety culture of healthcare, MMCs need to be structured in a standardized way to emphasize both patient safety and QI.

## Review

An integrative literature search was carried out using MEDLINE and PubMed. Combinations of the terms “morbidity”, “mortality”, “conference(s)”, or “rounds”, as well as “patient safety” were used in searches. The search was limited to human subjects and English articles only. This generated 228 articles. The titles and abstracts were then reviewed by all authors for relevance to the topic. Both quantitative and qualitative studies were included in the analysis. Those that had no mention of morbidity and mortality rounds or conferences, analyzed specific cases of morbidity or mortality, or concentrated primarily on the educational component without focus on patient safety or QI were not included in the analysis. Abstracts that were deemed relevant were read in their entirety. Further articles were identified through analysis of references and using the similar article function within the databases. This yielded a total of 21 articles for review. Articles were reviewed for content regarding structuring of the MMC to reflect and further develop QI and patient safety.

Three themes of QI-reflective MMC

The following three themes emerged that were consistently identified among the literature as being important for restructuring MMC to better reflect QI and patient safety: (1) the importance of careful case selection, (2) the format of discussion during the conferences, and (3) the action plans reflecting QI initiatives derived from the conferences.

Case Selection

The first stage of preparing for MMC is case selection. In the traditional model, there were no selection criteria for cases and they were often cases of mortality that were selected at random by a designated physician [5]. There was no clear guidance on case selection. However, the recurring theme yielded from the literature is that there should be a standardized method for case selection [6]. There should also be a mandatory reporting system in place, wherein cases are selected from this repository outlining systems failures with potentially preventable outcomes [5-10]. Studies to date stress the importance of not merely presenting a depiction of weird and wonderful cases, or cases in which death could be attributable solely to the disease process itself [9]. Furthermore, near misses, defined as events that have potential harm but are detected prior to reaching the patient, have particular value for discussion in these conferences from a patient safety and systems perspective [10,11].

With this emphasis on careful case selection, Tapper and Leffler [12] reported that case selection should be carried out at least four weeks prior to the MMC to allow ample time for preparation. From this, selection of cases should stem from system failures, allowing generation of productive discussion within the MMC itself. Recently, a study arising from the surgical discipline proposed a novel method of case selection, which could be readily incorporated into the selection process. They termed the new method “outcome expectedness”. Briefly, it requires all reported cases to be grouped into four categories to allow for better case selection. The four categories are expected successes, unexpected successes, expected failures, and unexpected failures. The focus, they propose, should not only be on unexpected failures but also on unexpected successes, as analyzing what led to these events could provide guidance in patient safety and QI initiatives in the future [13].

Format of Discussion

Once the case has been selected for presentation in MMC, it is the task of the presenter to convey a succinct version of this case, including all pertinent details, thereby facilitating the necessary elements for a discussion to follow. Traditionally, case presentation dominated the MMC, leaving little time for the discussion [5]. Throughout the literature, the proposed direction of the MCC is a movement toward a shorter presentation of the facts of the case through visual aids, e.g., a PowerPoint presentation, with minimal interruption from MMC attendees [14].

The case presentation is then followed by a discussion to identify system-wide issues evident in the case. There are two schools of thought on this discussion. One is that the presenter formulates the issues identified in the case prior to presentation and circulates a summary of these prior to discussion [11,14,15]. The second is that the generation of issues is a product of the MMC discussion itself, stressing the importance of involving other disciplines and healthcare professionals in an effort to identify all of the system failures demonstrated by the case [9,16,17]. The former allows for greater evaluation of the presenter’s understanding of the case with relation to QI and patient safety, and as the presenter is usually a medical resident, this allows for educational evaluation by attending physicians [11]. However, the latter allows for input across the healthcare professions, disciplines, and specialties, which has been shown previously to identify more issues needing improvement. Therefore, the latter would be the structure of choice with respect to advancing QI and patient safety [18].

In guiding the discussion component of the MMC, most studies describe a form of root cause analysis. This keeps the discussion goal-directed, which has been an issue in the past with more traditional forms of MMC [19]. Various forms have been proposed in the literature including ALARM (Association of Litigation and Risk Management) and the Ishikawa (or fishbone) diagram, which assigns all identified factors in the case to one of six categories: people, policy, procedure, equipment, environment, and other [5,10,14]. From this, Calder et al. [8] established the concept of bottom lines. Bottom lines outline the issues that are identified within the MMC and the lessons learned. Participants are then able to determine areas for improvement and formulate plans of action on their basis.

Identification and Implementation of QI Initiatives

Perhaps the most difficult aspect of restructuring MMC to better reflect patient safety and QI is the implementation of plans of action. These action plans or recommendations should be derived from the discussion of the issues identified following root cause analysis. The recommendations should be specific, and a plan should be detailed for implementation [8,20,21]. There should be a timeline outlined and a lead person identified to implement the specific plan [22]. One study advocated for the use of medical residents to receive assignment of a QI initiative stemming from MMC. It would then be their responsibility to implement the plan, seek assistance from other providers, and delegate tasks as necessary [7]. Participation in MMC should be sought from the hospital’s QI department to aid in planning and implementation. This would avoid the disconnect that is often seen between hospital QI departments and the frontline healthcare staff. It would further promote the collaboration of these two groups to work together to achieve results [10].

The importance of follow-up should not be understated. To implement change is one thing, but to follow through in monitoring is another entity where many MMCs routinely fail [22]. Some studies have suggested a dedicated 5 to 10 minutes at the beginning of every MMC outlining progress of previously developed action plans [6]. Others have advocated for a safety report bulletin summarizing meeting minutes of MMC and updates on progress of QI activities [9,14]. Evidently, ensuring sustainability of implemented changes by measuring and monitoring these changes for a long term is a major challenge in any QI initiative, as it requires the buy-in and cooperation and collaboration of the hospital and its healthcare professionals, which, in an already busy environment, can be seen as additional work.

Strengths

There are many strengths in the formulation of the three themes of QI reflective MMC. The studies were retrieved from multiple databases allowing for a more accurate depiction of the current literature on the topic. This included studies from many different sites as well as across various disciplines of medicine supporting its use. Most of the studies analyzed varied in proposals for the MMC, but the three themes - case selection, format of discussion, and identification and implementation of QI initiatives - remained consistent throughout the literature as primary pillars for QI and patient safety oriented MMC. The aspect that also remained consistent was the proposal for multidisciplinary and interprofessional involvement. This ensures a systems-based review versus that of individuals, as individual assessment would have less impact on QI in the healthcare setting. As depicted in a qualitative study by Szekendi et al. [23], this new setup would also foster a sense of community and teamwork within the healthcare setting, which could further improve communication and openness over time. This could also lead to improvement in patient safety itself.

In studies that analyzed outcomes with these three themes in place, there was a increase in attendance and participation in MMC. There was also improvement in the structure of MMC leading to the organization and implementation of QI initiatives. In a prospective observational study, Bal et al. [19] noted that while 91% of MMCs observed identified issues in the cases presented, a structured method for identification was used in less than 10%. Comparatively, by switching MMC from an education focus to that of QI, Tignanelli et al. [7] found that systems-based improvement strategies were identified in 73% of cases and that 82% of the proposed QI projects were successfully initiated. Similarly, a Canadian study implemented their own structured MMC model based on the three identified principles across disciplines in a single center and found 45 policy changes after the model was implemented, which was a stark contrast to zero that were implemented previously [24].

Weaknesses

One of the pivotal weaknesses of implementation of this model for restructuring the MMC is the fact that it is difficult to measure effectiveness. As specific adverse events identified through MMC are relatively uncommon occurrences, measuring their recurrence as a means of measuring effectiveness would be difficult to achieve. Assessment tools must be developed to monitor the changes arising from the MMCs. Kwok et al. [24] developed an assessment tool, the OM3 Scoring Index, to assess the effectiveness of implementation of the Ottawa Morbidity and Mortality Model (OM3) across 24 disciplines in one center. This tool was developed to measure the quality of MMC according to implementation of the key elements of OM3. Given that the pioneers of the OM3 created this assessment tool, there is potential for bias when using this as an assessment tool in other studies. Another indirect measure that has been suggested is following the action plans identified during the MMC to implementation and potential policy changes. The ratio of implemented to proposed changes could provide a surrogate marker for the QI and patient safety centered MMC effectiveness [10].

Another issue identified with the patient safety centered MMC is the time dedicated to the conference. Compiling and organizing all of the cases reported is more labor-intensive than simply recalling a recent case with a poor outcome. Furthermore, the presenter is tasked with the time-consuming process of choosing a case and collecting and organizing all of the information in a succinct manner [10]. Moreover, the conference itself would likely be more time-intensive with the extensive discussion and root cause analysis required in each session. With this proposed model of MMC, the work would not cease at the end of the session, but certain participants would be tasked with implementing and following up on action plans. This would add considerably to the already busy lifestyle of healthcare professionals, particularly medical residents with whom the majority of responsibility for these initiatives would likely lie [15].

Furthermore, de Vos et al. [22] identified that team dynamics may not have previously been considered. In their qualitative study, they determined through structured interviews with surgeons, surgical residents, and one physician assistant that although the involvement of other healthcare professionals may expand the discussion, it may also hinder it depending on the dynamics of that particular healthcare team. If the team is disjointed, participants may feel vulnerable and be unwilling to share opinions in the MMC setting [24]. This could be detrimental to the patient-centered, QI-focused MMC, which champions multidisciplinary participation.

Synthesis

As the patient safety centered and QI-focused MMC is a relatively new concept in the literature, further research in the area is needed. The consistent themes of case selection, format of discussion, and implementation of QI initiatives are repetitive in the literature and are emerging as the new era of MMC [6]. The importance of these themes cannot be overstated, and it is evident from the literature review that these are important concepts to follow if safety culture is to change through MMC. However, as depicted, there are no quantitative data to suggest that this new model results in measurable patient benefit through QI initiatives. Although this would be a difficult feat given the nature of the subject, it is important to have measurable outcomes when implementing such a drastic change. In addition, future studies should consider a longitudinal design. Studies may be able to delineate results on policy changes in the process of being implemented or changed but have not addressed the issue of maintaining these changes in the healthcare setting. This information can only come from monitoring and assessing over an extended period of time.

The proposed OM3 model piloted originally in the Emergency Medicine and Trauma departments in an Ottawa hospital offers a standardized way to institute MMC that is reflective of patient safety and QI [8]. This model was later expanded to 24 other disciplines within the same center with positive results [24]. It may be reasonable to consider implementation of this model for MMC in other healthcare settings across the world to investigate whether this model is transferable, and this could lead to international standardization of MMC.

## Conclusions

The MMC is a method of incident analysis that has been present in medicine for over a century. Given the new focus on patient safety and QI, the proposal is a shift from analysis of incidents that may or may not be preventable to those that have system-wide implications of failure, even in the face of no actual harm to the patient. This presents a major shift of focus that has only come about in the past decade. The three major themes associated with this change in focus include careful case selection, format of discussion, and the resulting QI initiatives and their implementation. In addition, a multidisciplinary approach and inclusion of hospital QI personnel can lead to further identification of patient safety issues and areas for improvement. It may further serve to foster a new safety culture for the healthcare teams involved as well. The result is QI initiatives and policy changes, but the ongoing issue focuses on the inability to measure the effectiveness of these changes to the MMC format. Also, time constraints and the labor-intensive nature of the new form of MMC have also been cited as potential weaknesses in the system. Evidently, further research should focus on instituting measures of effectiveness for these new MMC models, as well as perhaps leading to one standardized method of MMC implementation that encompasses the three pivotal themes.
